# Epithelial cells with high TOP2A expression promote cervical cancer progression by regulating the transcription factor FOXM1

**DOI:** 10.3389/fonc.2025.1604960

**Published:** 2025-06-16

**Authors:** Wei Sun, Lu Chen, Xiaoling Feng

**Affiliations:** ^1^ Department of Gynecology II, First Affiliated Hospital of Heilongjiang University of Chinese Medicine, Harbin, China; ^2^ Siping City Central People's Hospital, Siping, China

**Keywords:** epithelial cells, cervical cancer, FOXM1, single-cell RNA sequencing, TOP2A

## Abstract

**Background:**

Cervical cancer (CC) remains a major malignancy threatening women’s health, with high-grade squamous intraepithelial lesions playing a critical role in the progression toward CC. Exploring the molecular characteristics of epithelial cells (EPCs) as high-stage intraepithelial neoplasia evolves into CC is essential for the development of effective targeted drugs for cervical cancer. Single-cell RNA sequencing technology can fully understand the immune response at each molecular level, providing new ideas and directions for the precise treatment of CC.

**Methods:**

Single-cell RNA sequencing was employed to comprehensively map EPCs characteristics. The differentiation trajectory of EPCs was inferred using Slingshot, while enrichment analysis highlighted the biological functions of EPCs. Cellchat visualized cell-cell interactions, and SCENIC was used to infer transcription factor regulatory networks in EPCs. CCK-8, colony formation, and EDU experiments were used to verify cell proliferation changes. Scratch assays and transwell assays were used to verify cell migration and invasion.

**Results:**

A distinct EPCs subpopulation with high *TOP2A* expression was identified, predominantly originating from tumor tissues. This subpopulation exhibited disrupted mitosis and cell cycle regulation, along with features of high proliferation, high energy metabolism, and matrix plasticity. It played a key role in shaping the tumor microenvironment via the LAMC1-(ITGA3-ITGB1) signaling pathway. FOXM1, a key transcription factor in this cell subpopulation, significantly inhibited the proliferation and invasion of cervical cancer cells.

**Conclusion:**

Through in-depth analysis of EPCs, this study provides promising insights and potential therapeutic targets for precision targeted treatment strategies for CC.

## 1 Introduction

Cervical cancer (CC) ranks among the most prevalent gynecological malignancies and is the fourth leading cause of cancer-related deaths in women, marked by high morbidity and mortality rates (1–3). Despite significant improvements in survival rates and quality of life for early-stage patients through a combined treatment approach of surgery, radiotherapy, and chemotherapy, some patients still experience drug resistance or disease progression. With the advancement of modern medicine, finding new targets for malignant tumors has become a hot issue in immunotherapy, precision therapy and reversing tumor immune resistance (4–7). Immunotherapy and targeted therapies offer promising alternatives for patients with recurrent or metastatic CC, with the potential to deliver more precise and effective treatment options (8).

In the progression of CC, cervical intraepithelial neoplasia (CIN) represents a critical turning point. While most low-grade CIN lesions can regress spontaneously, high-stage intraepithelial neoplasia (HSIL) poses a significantly higher risk of malignant transformation. Thus, investigating the core molecular mechanisms underlying the progression from HSIL to CC is essential for advancing early prevention strategies for cervical cancer (9–11).

The tumor microenvironment is a protective chamber that maintains the occurrence and development of tumors. Epithelial cells (EPCs), as the most common origin of tumor cells, play a vital role in tumor progression. As a type of stromal cells, EPCs are also able to support and maintain chronic inflammatory states and immune resistance in the tumor microenvironment. Therefore, treatment targeting EPCs may provide a promising approach for immune resistance in cervical cancer.

Multi-omics technologies technology has been widely used in cancer cell biology and molecular biology related research (12–20) especially in the field of immune resistance (8, 14, 20–27). This study used Single-cell RNA sequencing(scRNA-seq) to deeply explore the map characteristics of EPCs during CC progression, explained the biological characteristics of EPCs from the level of transcriptional regulatory network, and identified key pathogenic EPCs subpopulations. Our research provides potential cell vectors and molecular targets for the precise targeted treatment of CC, and provides a practical idea for the development of CC anti-tumor drugs.

## 2 Methods

### 2.1 Data source

Single-cell sequencing data were obtained by accessing the ArrayExpress (https://www.ebi.ac.uk/biostudies/arrayexpress) database with CC number E-MTAB-12305. The research data were derived from public databases, so no ethical approval was required.

### 2.2 Data quality control

The raw data were imported into R software (version 4.3.2) using Seurat R package for analysis. The DoubletFinder function (28–30) was used to perform strict quality control on the scRNA-seq data to filter low-quality cells and remove potential doublet cells. The quality control standards were as follows: 300<nFeature<6000; 500<nCount<50000; 0<pMT<25; 0<pHB<5.

The data were normalized using the NormalizeData function (31–35). The FindVariable Features function was used to find highly variable genes (36–40). The ScaleData R package was used for standardization processing of the data. Principal component analysis (PCA) (41–43) was performed on the data, and the top 30 principal components were selected. The harmony R package (44, 45) was used to remove batch effects.

### 2.3 Cell identification

The cells were clustered using the FindClusters function and the FindNeighbors function (28, 46), and the cells were annotated according to the CellMarker database (http://xteam.xbio.top/CellMarker/) and cell-typical markers. The FindAllMarkers function was used to find differentially expressed genes in different cell subpopulations.

### 2.4 InferCNV

Taking ECs as reference, the inferCNV algorithm was used to calculate the copy number variation of EPCs.

### 2.5 Enrichment analysis

Gene ontology (GO)enrichment analysi (47–52) of differentially expressed genes was performed using ClusterProfiler R package (51, 53, 54). Gene set enrichment analysis (GSEA)was performed by downloading GSEA software from the GSEA website (http://software.broadinstitute.org/gsea/msigdb).

### 2.6 Slingshot analysis

The getlineage function in the Slingshot R package is used to infer cell differentiation lineages (55–57). The getCurves function is used to calculate the cell expression levels of different lineages within the fitting time.

### 2.7 CellChat analysis

CellChat R package (58–60) was used to infer the interactions between cells. The identifyCommunicationPatterns algorithm is used to count the number of communication patterns, and the netVisual_diffInteraction algorithm is used to calculate the difference in the strength of communication between cells. Circle plots, violin plots, layer plots, and heat maps are used to visualize the communication between cells.

### 2.8 Scenic analysis

The activity of transcription factors in EPCs subsets was assessed using the SCENIC package in Python (version 3.7). The GRNBoost2 algorithm was used to infer co-expression modules between transcription factors and target genes. The RcisTarget algorithm was used to analyze the genes in each co-expression module to help identify enriched motifs. The AUCell score was used to evaluate the activity of transcription factors in EPCs.

### 2.9 Cell transfection

HeLa cell lines and SiHa cell lines were propagated in MEM medium. Cells were seeded at 50% density in 6-well plates and then transfected with FOXM1-specific knockdown constructs (si-*FOXM1–*1 and si-*FOXM1*-2) and negative control constructs (si-NC). Transfection was performed using Lipofectamine 3000RNAiMAX (Invitrogen, USA) according to the manufacturer’s instructions. siRNA sequences: si-1: AAGAAGAAAUCCUGGUUAA; si-2: ACUAUCAACAAUAGCCUAU. qRT-PCR primers: F: AAACCTGCAGCTAGGGATGT; R: AGCCCAGTCCATCAGAACTC.

### 2.10 CCK-8 assay

After being plated in 96-well plates, the cells were cultured for a full day. After adding 10 μL of CCK-8 labeling reagent to each well, the wells were left in the dark for two hours. Using an enzyme marker, absorbance at 450 nm was used to measure cell viability.

### 2.11 Transwell assay

After the cells were starved for twenty-four hours, the cell suspension was combined with Matrigel and introduced onto the Costar plate’s upper chamber. The lower chamber was filled with the serum-containing media. The cells were fixed with 4% paraformaldehyde and stained with crystal violet following a 48-hour incubation period.

### 2.12 Wound healing assay

The cell monolayer in each well of a six-well plate was consistently scratched using a sterile 200 μL pipette tip, and scratch photos were recorded after 0 and 48 hours of incubation. Scratch width was measured using Image-J software.

### 2.13 5-Ethynyl-2’-deoxyuridine proliferation assay

After adding the EdU working solution, the cells were incubated for two hours. Following a PBS wash, the cells were fixed using 4% paraformaldehyde solution, permeabilized and quenched using a solution containing 0.5% Tr and 2 mg/ml glycine, and stained using 1X Apollo solution and Hoechst staining reaction solution.

### 2.14 Statistical analysis

All data were processed using R or Python. P< 0.05 was considered statistically significant.

## 3 Results

### 3.1 Single-cell transcriptome profiles during CC progression

**Figure 1:**
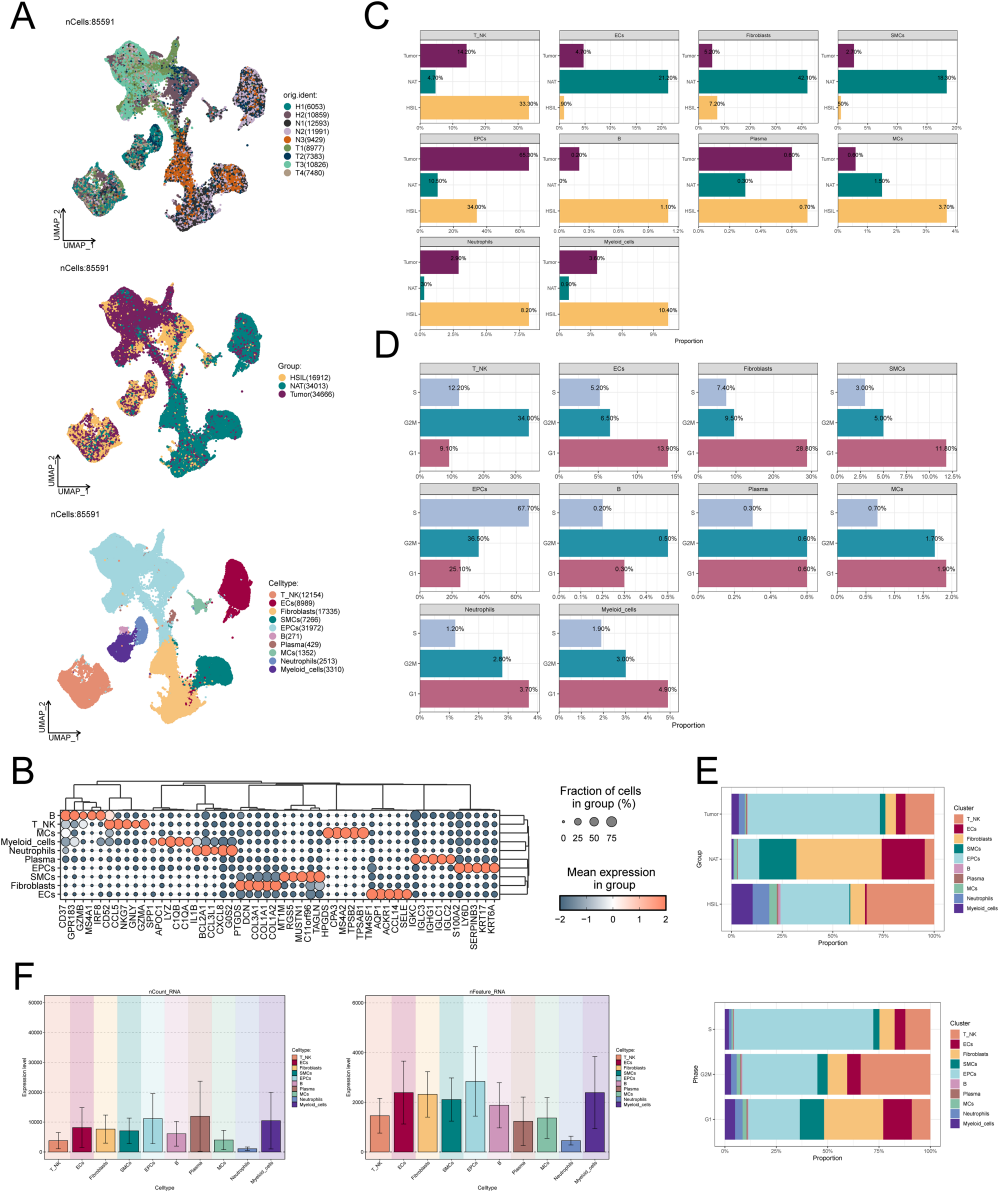
scRNA-seq characterizes the cellular landscape of CC. **(A)** Dimensionality reduction clustering diagram of 85,591 high-quality cells. From top to bottom, the sample sources, sample groups (HSIL, NAT and tumor) and cell types (T cells and NK cells, ECs, fibroblasts, SMCs, EPCs, B cells, plasma cells, MCs, neutrophils and myeloid cells) of all cells wereshown. **(B)** Top 5 marker genes of 10 cell types. The bubble size represents the Fraction of cells in group, and the bubble color represents the average expression level. **(C-E)** The proportion of 10 cell types in different samplegroups (HSIL, NAT and tumor) and different cell cycle phases (G1, G2M and S). **(F)** nCount-RNA and nFeature-RNA expression in 10 celltypes. nFeature_RNA: The number of different genes detected in each cell. nCount_RNA: The total number of all RNA molecules sequenced in each cell.

To explore the evolution of transcriptome features during CC development, we performed scRNA-seq analysis on 9 samples from normal cervix (NAT), HSIL and cervical cancer (Tumor), and finally identified 85,591 high-quality cells, as shown in **Figure 1A**. Using known cell type marker genes (**Figure 1B**), we finally identified 10 types of cells in these samples, including T cells and NK cells, ECs, fibroblasts, smooth muscle cells (SMCs), EPCs, B cells, plasma cells, mast cells (MCs), neutrophils and myeloid cells As shown in **Figure 1C**, EPCs are the main cell type present in tumor samples, accounting for 65.3%, and are mainly in the G2M and S phases of the cell cycle, with active cell replication and vigorous proliferation (**Figure 1D**). **Figure 1E** confirms this result. In addition, we also looked at the number of molecules and total number of genes detected in different cell types, and the results showed that EPCs had higher nCount-RNA and nFeature-RNA expression (**Figure 1F**). This suggests that EPCs have higher cell activity and rich gene expression diversity.

### 3.2 EPCs are heterogeneous during CC progression

**Figure 2:**
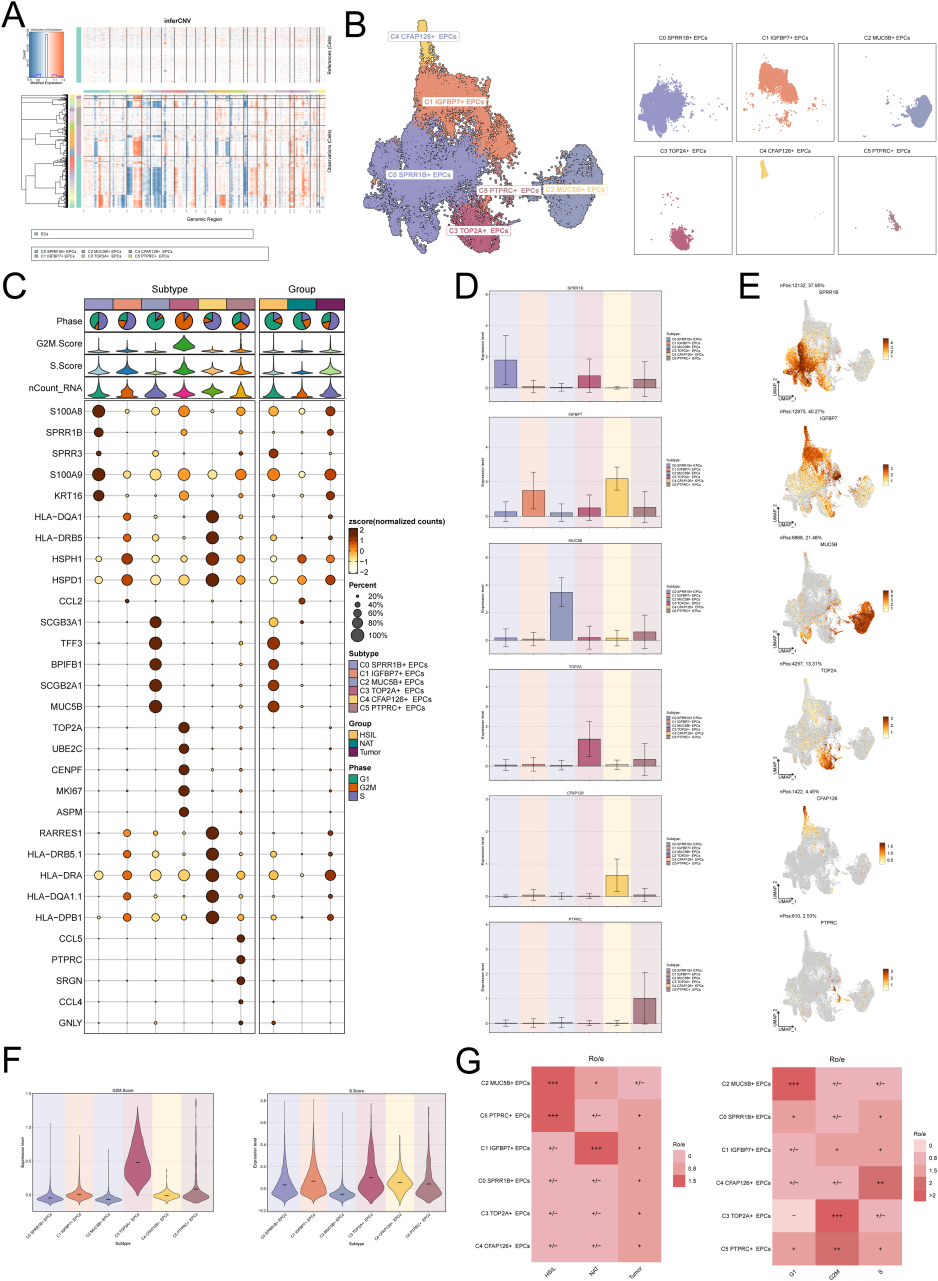
Heterogeneity profile of EPCs within CC. **(A)** inferCNV is used to infer the copy number variation level of EPCs. **(B)** Clustering and faceting diagrams of the six EPCs subtypes. **(C)** Expression levels of the top 5 markers of the six EPCs subtypes in different EPCs subtypes and sample source groups. The bubble size represents the expression percentage, and the bubble color represents the zscore. **(D, E)** The bar graph and umap graph show the expression levels of the named genes of the six EPCs subtypes. **(F)** G2M.score and S.score of the six EPCs subtypes. **(G)** Ro/e of the six EPCs subtypes in different sample groups (HSIL, NAT and tumor) and different cell cycle phases (G1, G2M and S).

As the precursor of tumor cells, the molecular characteristics of EPCs during cancer evolution are crucial to reveal the origin and development of tumors. Therefore, we conducted an in-depth exploration of the characteristics of EPCs during CC progression. First, we used inferCNV to analyze EPCs to view their copy number variations (**Figure 2A**). Next, we re-clustered EPCs and identified 6 EPCs subtypes: C0 *SPRR1B*+ EPCs, C1 *IGFBP7*+ EPCs, C2 *MUC5B*+ EPCs, C3 *TOP2A*+ EPCs, C4 *CFAP126*+ EPCs, and C5 *PTPRC*+ EPCs (**Figure 2B**). **Figure 2C** shows the expression of the top 5 marker genes of the 6 EPCs subtypes and different groups. The expression levels of the named genes of the 6 EPCs subtypes are shown in bar graphs and UMAP graphs (**Figures 2D, E**). It is worth mentioning that we found that C3 *TOP2A*+ EPCs had the highest expression of G2M and S phase cell cycle phase scores (**Figure 2F**), which means that this EPCs subpopulation has active proliferation and may play an important role in the rapid progression of tumors. In-depth analysis found that C3 *TOP2A*+ EPCs tend to be distributed in tumor samples (**Figure 2G**), suggesting that this EPCs subpopulation may be tumor-related EPCs.

### 3.3 C3 TOP2A+ EPCs are the key EPCs subset for CC progression

**Figure 3:**
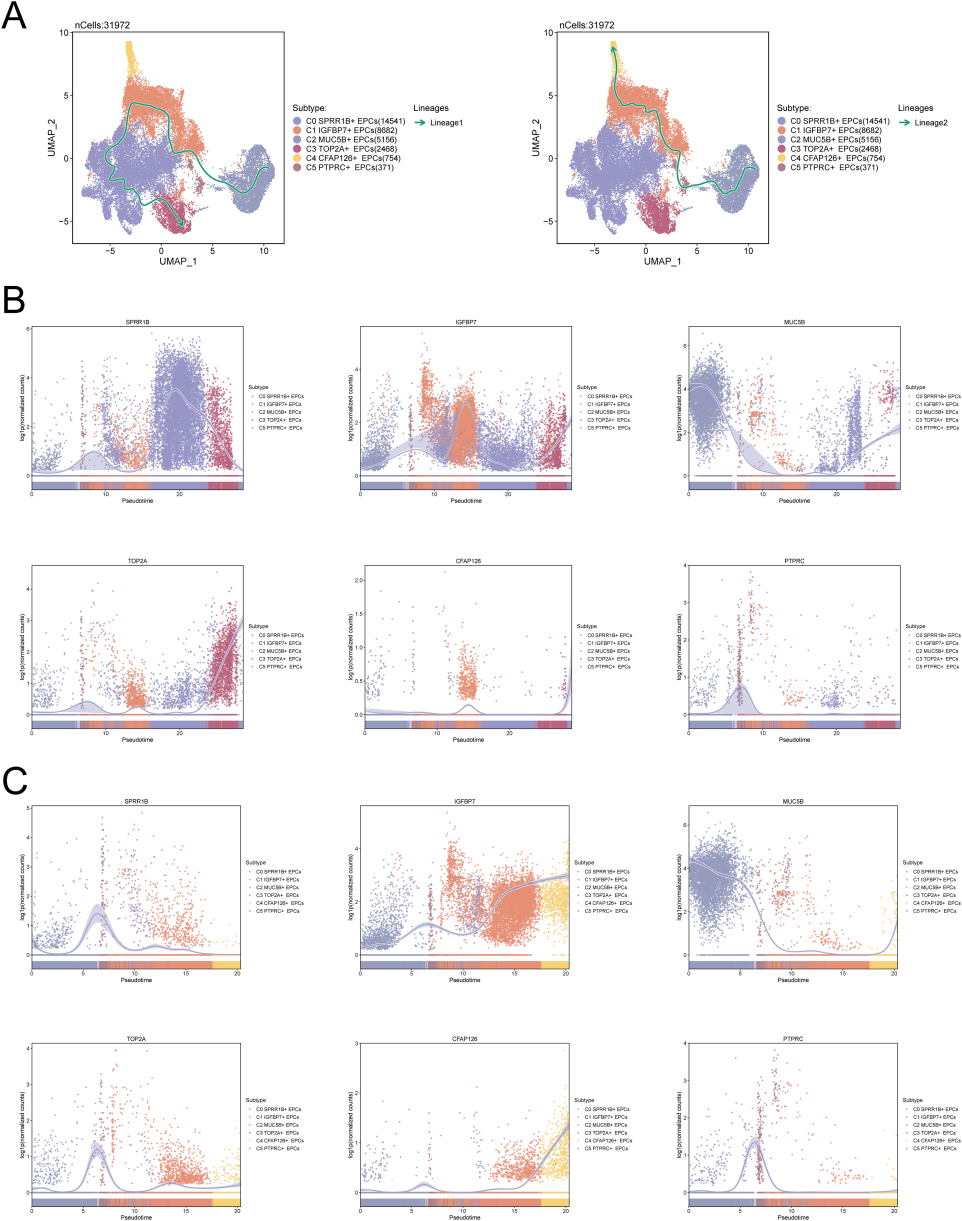
C3 *TOP2A*+ EPCs are located at the end of the EPCs differentiation trajectory. **(A)** Slingshot predicts two differentiation lineages of EPCs (left: lineage 1, right: lineage 2). The solid line represents the cell differentiation trajectory, and the arrow direction represents the transition of cell differentiation from immature to mature. **(B, C)** Dynamic trajectory diagram of the expression levels of the six EPCs subpopulation named genes predicted by Slingshot in the two differentiation lineages of EPCs over time (B: lineage 1, C: lineage 2).

To verify the above results, we used Slingshot to infer the differentiation trajectory of EPCs. As shown in **Figure 3A**, in the differentiation lineage 1 of EPCs, C3 *TOP2A*+ EPCs are located at the end of the differentiation trajectory, which is consistent with the results of **Figure 2G**, that is, the PCs subpopulation is tumor-associated EPCs and is of key significance in the evolution of CC. While in lineage 2, C4 *CFAP126*+ EPCs are located at the end. **Figures 3B, C** show the differences in the expression levels of the named genes of the six EPCs subtypes over time, among which *TOP2A* is mainly expressed at the end of lineage 1.

### 3.4 C3 TOP2A+ EPC regulates mitosis and energy metabolism

**Figure 4:**
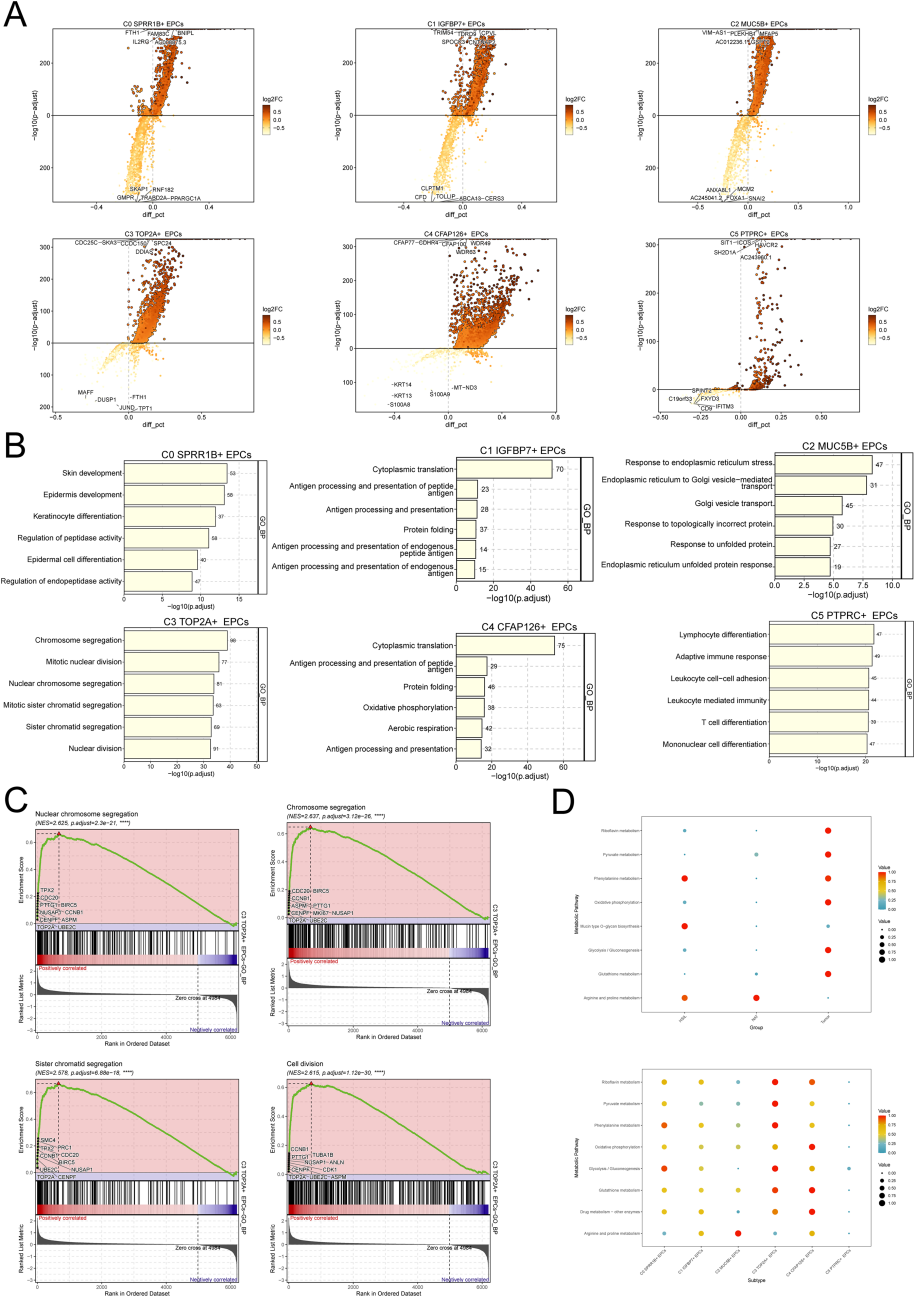
Heterogeneity of biological characteristics of different EPCs subgroups. **(A)** Differential gene analysis of 6 EPCs subtypes. The top 5 genes with upregulated differential expression are shown above the horizontal line, and the top 5 genes with downregulated differential expression are shown below the horizontal line. **(B)** GO-BP enrichment analysis of 6 EPCs subgroups. **(C)** GSEA results of C3 *TOP2A*+ EPCs. **(D)** Metabolic pathway enrichment analysis of EPCs.

To further explore the biological characteristics of C3 *TOP2A*+ EPCs, we performed differential gene analysis and enrichment analysis on different subtypes of EPCs (**Figures 4A, B**). As shown in the figure, C3 *TOP2A*+ EPCs are mainly enriched in chromosome segregation, mitotic nuclear division, nuclear chromosome segregation, mitotic sister chromatid segregation, sister chromatid segregation and nuclear division-related pathways, which are closely related to chromosome division and mitosis. In addition, GSEA further confirmed the above findings (**Figure 4C**). It is worth mentioning that with the progression of CC, the metabolic activity of EPCs gradually increased, especially in pathways such as riboflavin metabolism, pyruvate metabolism, oxidative phosphorylation, and glycolysis/gluconeogenesis. Consistent with this, the metabolic activity of these pathways was also significantly enhanced in C3 *TOP2A*+ EPCs (**Figure 4D**).

### 3.5 C3 TOP2A+ EPCs interact with other cells via the LAMC1-pathway

**Figure 5:**
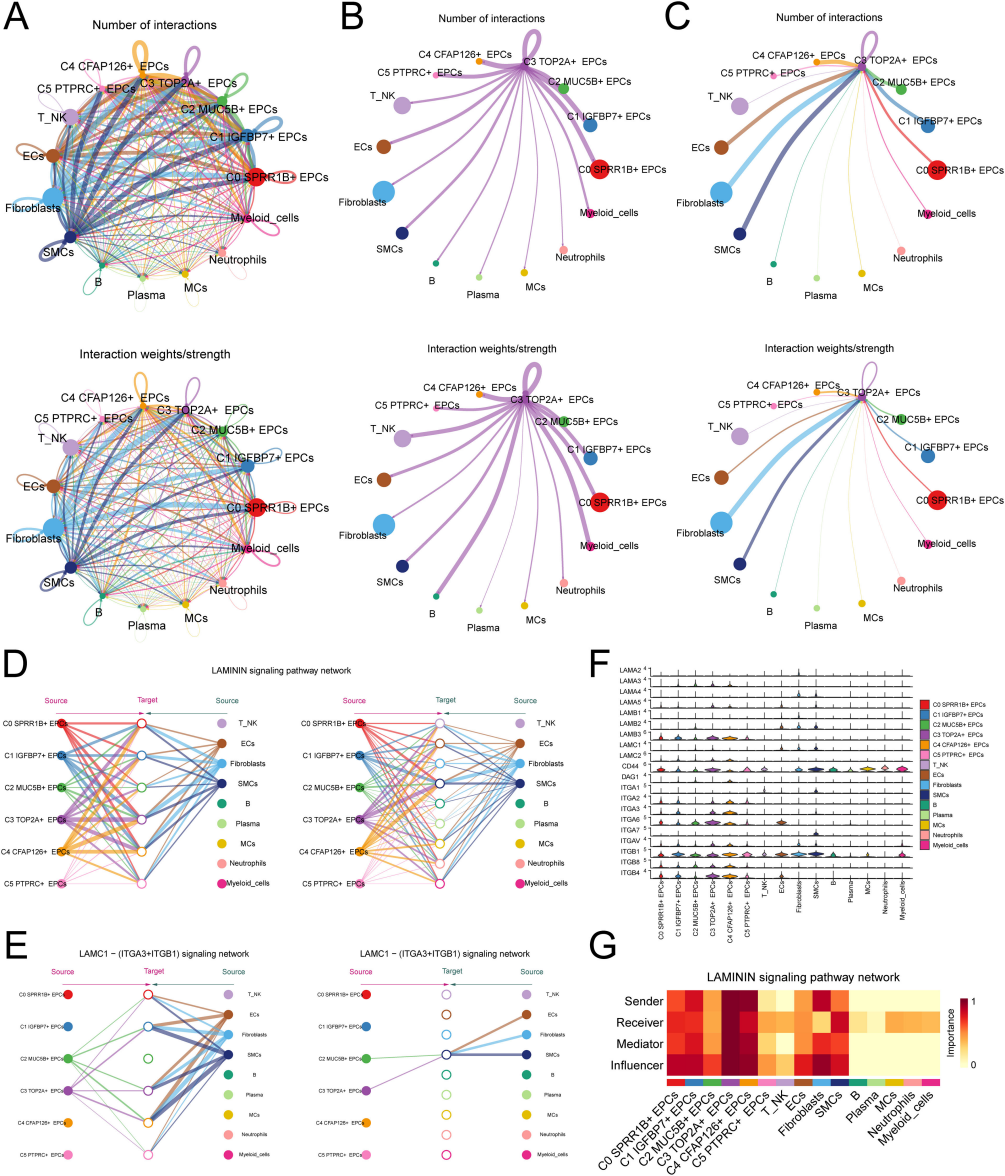
Cell interaction analysis of C3 *TOP2A*+ EPCs. **(A)** The number and weight of interactions between EPCs subtypes and other cell types. **(B)** Circle diagram of the number and weight of interactions with C3 *TOP2A*+ EPCs as signal senders and other cell types as signal receivers. **(C)** Circle diagram of the number and weight of interactions with C3 *TOP2A*+ EPCs as signal receivers and other cell types as signal senders. **(D)** A diagram of the communication hierarchy between cell types in the LAMININ signaling pathway. **(E, F)** Hierarchical diagram and violin plots of communication between cell types in the LAMC1 - (ITGA3+ITGB1) ligand receptor pathway. **(G)** Heat map of the centrality of communication between cells in the LAMININ signaling pathway.

To reveal the crosstalk relationship between C3 *TOP2A*+ EPCs and other cells, we first analyzed the number and strength of interactions between EPCs and other cell types using cellular communication (**Figure 5A**). Further research found that when C3 *TOP2A*+ EPCs serve as signal senders, they have a strong interaction with immune cells, such as T cells and NK cells, B cells and myeloid cells, suggesting that they are closely related to the shaping of the tumor immune microenvironment (**Figure 5B**). When C3 *TOP2A*+ EPCs serve as signal receptors, they interact strongly with stromal cells, such as fibroblasts and SMCs, affecting the matrix shaping of the tumor microenvironment (**Figure 5C**). This suggests that C3 *TOP2A*+ EPCs have significant plasticity.

Analysis found that C3 *TOP2A*+ EPCs interacted with other cells mainly through the LAMININ signaling pathway, especially the LAMC1 - (ITGA3+ITGB1) ligand receptor pair (**Figures 5D-F**). In this pathway, C3 *TOP2A*+ EPCs mainly interact with immune cells such as T-NK cells and myeloid cells through paracrine effects. Finally, we used centrality scores to visualize the role of C3 *TOP2A*+ EPCs in this pathway (**Figure 5G**).

### 3.6 Transcription factor regulatory network of EPCs

**Figure 6:**
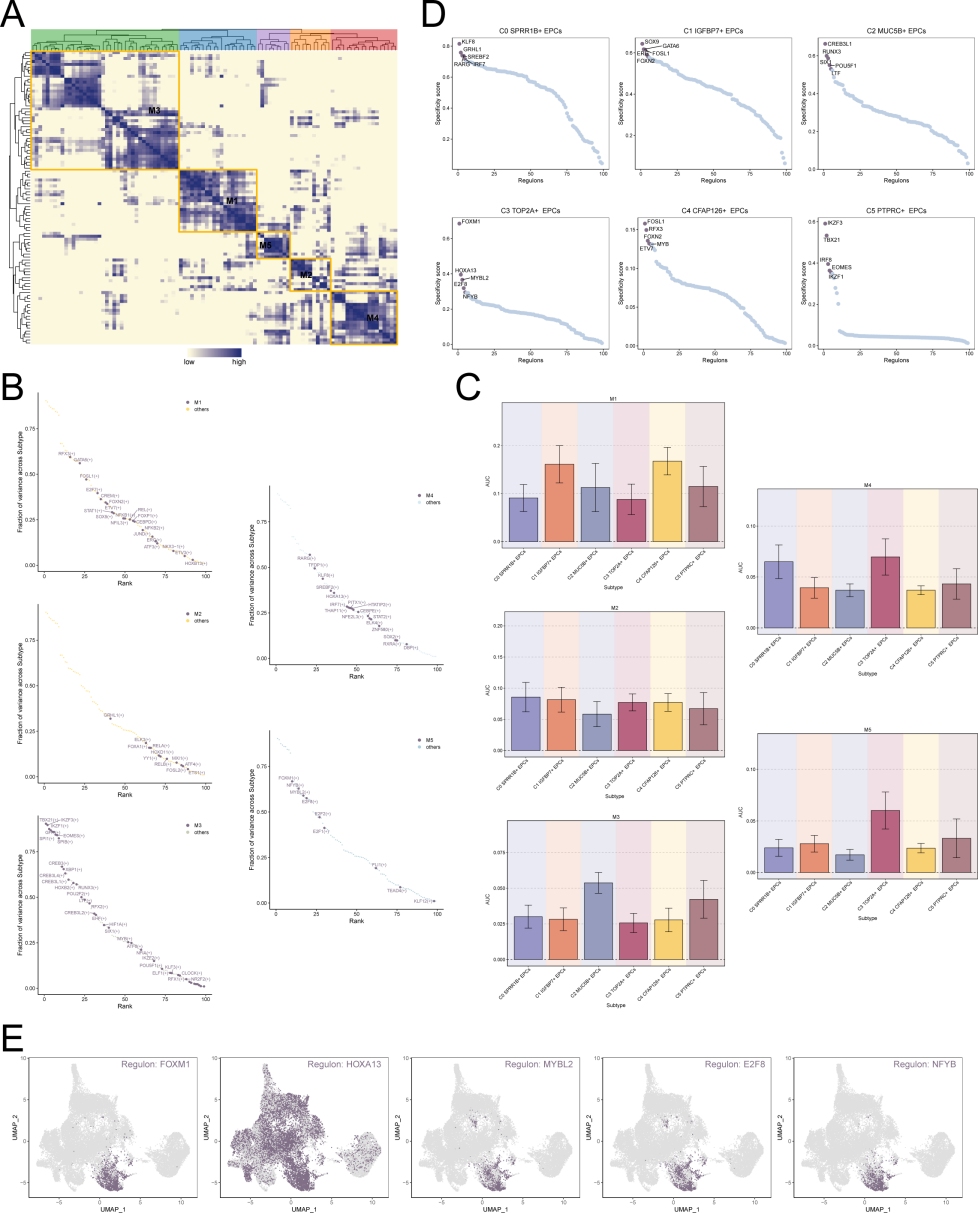
Transcription factor regulatory network of EPCs. **(A)** Based on the connection specificity index matrix, 5 regulatory modules of TAMs subtypes were identified. **(B)** In EPCs subtypes, the regulators in different regulatory modules were ranked based on variance scores. **(C)** Expression of different regulatory modules in EPCs subtypes. **(D)** Ranking of top 5 transcription factors in different EPCs subtypes based on regulator specificity score. **(E)** Expression of top 5 key regulators FOXM1, HOXA13, MYBL2, E2F8 and NFYB of C3 *TOP2A*+ EPCs in all EPCs.

Transcription factors can act directly on the genome, regulate gene transcription by binding to specific nucleotide sequences upstream of genes, and affect the biological function of cells. Therefore, we used the scenic algorithm to analyze the gene regulatory network of EPCs. Different transcription factors can jointly regulate the expression of certain genes. Therefore, we divided the transcription factors of EPCs into 5 modules based on the connection specificity index: M1, M2, M3, M4 and M5 (**Figure 6A**). As shown in **Figures 6B, C**, the transcription factors in the M4 and M5 modules may play a major regulatory role in the biological characteristics of C3 *TOP2A*+ EPCs. Further analysis of the top 5 transcription factors of different EPCs subtypes revealed that the transcription factors of C3 *TOP2A*+ EPCs were FOXM1, HOXA13, MYBL2, E2F8 and NFYB (**Figures 6D, E**). This is consistent with previous studies showing that E2F8 can maintain tumor cell proliferation and promote tumor cell migration (61, 62). Among them, FOXM1, as an important oxidative stress response regulator and proliferation-related factor, has a significant impact on the progression and outcome of tumors.

### 3.7 FOXM1 is a potential therapeutic target for CC

**Figure 7:**
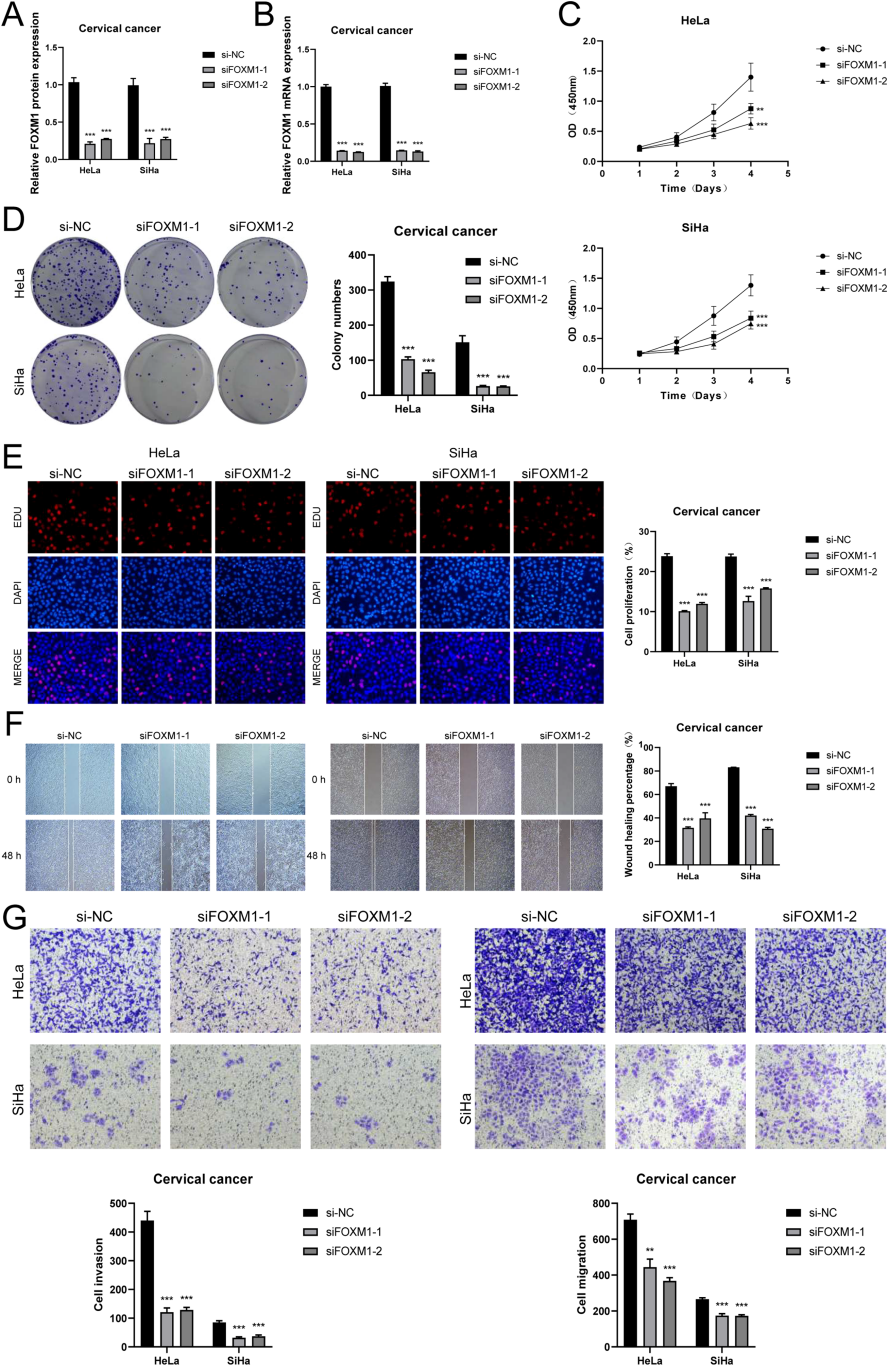
*FOXM1* knockdown inhibits cervical cancer cell proliferation and invasion. **(A, B)** After *FOXM1* knockdown, its mRNA and protein expression abundance were significantly reduced. **(C)** CCK-8 showed changes in cervical cancer cell activity after *FOXM1* knockdown. **(D, E)** Colony formation and EDU showed that cell proliferation slowed down after *FOXM1* knockdown. **(F)** Scratch assay and quantitative analysis of FOXM1 knockdown. **(G)** Transwell assay and quantitative analysis of *FOXM1* knockdown. (**P < 0.01, ***P < 0.001.).

To verify the key role of *FOXM1* in cervical cancer, we knocked down *FOXM1* in CC cell lines (**Figures 7A, B**). CCK-8 showed that after *FOXM1* knockdown, the cell viability of cervical cancer cells decreased significantly (**Figure 7C**), and cell proliferation slowed down (**Figure 7D**). The EDU experimental results are consistent with this (**Figure 7E**). The results of the scratch experiment and transwell experiment showed that the reduced expression of *FOXM1* significantly inhibited the migration and invasion ability of CC cells (**Figures 7F, G**). Based on the above results, *FOXM1* is a key target for inhibiting the progression and invasion of CC and a potential target for future clinical drug development.

## 4 Discussion

Blocking the formation of the tumor microenvironment is of great significance for the early prevention and diagnosis and treatment of cancer (63–65). CIN is an important turning point in the development of CC (66, 67). We used scRNA-seq technology to comprehensively characterize the changes in the intracellular molecular profiles of normal tissues developing into HSIL and eventually evolving into CC. Tumor cells (especially epithelial malignant tumors) are usually derived from the malignant transformation of normal epithelial cells. Epithelial cells cover the body surface or form glands, and have polarity, tight arrangement, and reliance on intercellular connections under physiological conditions. Malignant epithelial cells (tumor cells) lose these characteristics and gain invasiveness and metastasis capabilities. Therefore, revealing the molecular biological characteristics of epithelial cells during cancer evolution is a core hotspot for studying tumor precision treatment strategies.

We identified a type of EPCs with high proliferation characteristics that mainly exist in tumor tissues, and further explored the cell interaction crosstalk and transcription factor regulatory network of this type of EPCs. Experiments confirmed that the transcription factor FOXM1 is a promising target for CC treatment. This study provides reference significance for the interception and treatment of CC, and also provides potential targets for the development of anti-tumor drugs.

TOP2A is a key subtype of DNA topoisomerase, and the DNA breaks mediated by it are of great significance for the carcinogenesis of normal cells (68). Studies have found that TOP2A can interact with RNA Pol II on mitotic chromosomes to restart transcription, promote the mitotic process, and ensure cell proliferation and growth (69). In addition, this type of EPCs subtype highly expresses cell cycle-related genes such as *UBE2C*, *CENPF*, and *MKI67 (* 70, 71), explaining the gene characteristics behind the high proliferation function of C3 *TOP2A*+ EPCs. Slingshot analysis confirmed the finding that EPCs gradually evolved from normal cells to tumor-related EPCs, which is consistent with the sample composition in this study, that is, the development of CC goes through three stages: normal tissue, HSIL, and tumor tissue. Combined with the results of enrichment analysis, the rapid proliferation of malignant EPCs in the tumor stage greatly accelerates the progression and invasion of CC.

Studies have found that increased glycolysis and enhanced oxidative stress response under hypoxic conditions can accelerate the proliferation of EPCs. This provides support for the high metabolic activity of C3 *TOP2A*+ EPCs and reveals the connection between EPCs and energy metabolism in CC.

The activation of laminin and integrin-related pathways creates favorable conditions for matrix shaping of the tumor microenvironment and immune escape of tumor cells. Previous studies have found that LAMC1, or laminin γ1, is a biomarker for CC prognosis (72, 73). Our study confirms this result and reveals its intrinsic molecular mechanism for promoting the progression of cervical cancer. LAMC1 binds to integrin (ITGA3-ITGB1) receptors, inducing the activation of the laminin signaling pathway, further leading to the formation of extracellular matrix, hindering the recruitment of immune effector cells into the TME to exert anti-cancer effects. Therefore, breaking the matrix niche and restoring normal immune response is the only way to avoid immune resistance and exert anti-tumor effects.

*FOXM1*, a forkhead box gene, plays an important role in cell proliferation and lifespan and has been considered as a potential target for CC treatment in recent years. FOXM1 is one of the main transcription factors that regulates PD-L1 expression and ICI immune response in tumors, and can increase the sensitivity of tumor cells to immunotherapy. Studies have found that FOXM1-mediated inactivation of inflammasome transcription can promote the immunosuppressive microenvironment of CC and accelerate the immune escape of cancer cells (74). Our study not only provides single-cell and experimental evidence for the treatment of cervical cancer with *FOXM1*, but also suggests that there may be a potential connection between *FOXM1* and *TOP2A*, but this hypothesis still needs to be verified experimentally (75). Previous studies have found that the co-expression of FOXM1 and TOP2A is significantly associated with poor prognosis in patients with colorectal cancer, bladder cancer, etc (76, 77). In addition, both can accelerate tumor immune escape and immunosuppression. At the same time, the close relationship between tumor proliferation and tumor cell extracellular matrix destruction still needs further exploration. Finally, the limited amount of sample data may lead to the lack of universality of our study.

## 5 Conclusions

In summary, our study provides a comprehensive and in-depth map of the molecular mechanism of EPCs in CC through scRNA-seq. EPCs with high expression of *TOP2A* are expected to become a key cell subpopulation for the treatment of CC in the future. On this basis, the development of anti-tumor drugs *FOXM1* inhibitors will also bring hope for the diagnosis and treatment of CC.

## Data Availability

The original contributions presented in the study are included in the article/supplementary material. Further inquiries can be directed to the corresponding author.

## References

[1] Huang M Chen M Qi M Ye G Pan J Shi C. Perivascular cell-derived extracellular vesicles stimulate colorectal cancer revascularization after withdrawal of antiangiogenic drugs. J Extracell Vesicles. (2021) 10:e12096. doi: 10.1002/jev2.12096 34035882 PMC8138700

[2] Fotopoulou C Khan T Bracinik J Glasbey J Abu-Rustum N Chiva L. Outcomes of gynecologic cancer surgery during the COVID-19 pandemic: an international, multicenter, prospective CovidSurg-Gynecologic Oncology Cancer study. Am J Obstet Gynecol. (2022) 227:731–5. doi: 10.1016/j.ajog.2022.06.052 PMC9242690 35779589

[3] Do MH Shi W Ji L Ladewig E Zhang X Srivastava RM. Reprogramming tumor-associated macrophages to outcompete endovascular endothelial progenitor cells and suppress tumor neoangiogenesis. Immunity. (2023) 56:2555–69. doi: 10.1016/j.immuni.2023.10.010 PMC11284818 37967531

[4] Yang L Wang Q He L Sun X. The critical role of tumor microbiome in cancer immunotherapy. Cancer Biol Ther. (2024) 25:2301801. doi: 10.1080/15384047.2024.2301801 38241173 PMC10802201

[5] Li Z Liu Y Huang X Wang Q Fu R Wen X. F. Nucleatum enhances oral squamous cell carcinoma proliferation via E-cadherin/beta-Catenin pathway. BMC Oral Health. (2024) 24:518. doi: 10.1186/s12903-024-04252-3 38698370 PMC11064238

[6] Xu Y She Y Li Y Li H Jia Z Jiang G. Multi-omics analysis at epigenomics and transcriptomics levels reveals prognostic subtypes of lung squamous cell carcinoma. BioMed Pharmacother. (2020) 125:109859. doi: 10.1016/j.biopha.2020.109859 32036209

[7] Zhu Y Liang L Zhao Y Li J Zeng J Yuan Y. CircNUP50 is a novel therapeutic target that promotes cisplatin resistance in ovarian cancer by modulating p53 ubiquitination. J Nanobiotechnol. (2024) 22:35. doi: 10.1186/s12951-024-02295-w PMC10799427 38243224

[8] Tan L Wu S Qiu Y Jie Y Zhang S Zhou S. Preliminary investigation into ultrasound and MRI presentation of large-cell neuroendocrine carcinomas of the uterine cervix. Bio Integrat. (2023) 4(4):180–185. doi: 10.15212/bioi-2022-0028

[9] Xiao X Wang W Bai P Chen Y Qin Z Cheng T. Genomic imprinting biomarkers for cervical cancer risk stratification. Cancer Commun (Lond). (2024) 44(12):1385–1390. doi: 10.1002/cac2.v44.12 PMC11666962 39388677

[10] Wang J Elfstrom KM Dillner J. Human papillomavirus-based cervical screening and long-term cervical cancer risk: a randomised health-care policy trial in Sweden. Lancet Public Health. (2024) 9:e886–95. doi: 10.1016/S2468-2667(24)00218-4 39486904

[11] Jana D Zhao Y. Strategies for enhancing cancer chemodynamic therapy performance. Explor (Beijing). (2022) 2:20210238. doi: 10.1002/EXP.20210238 PMC10191001 37323881

[12] Ye B Fan J Xue L Zhuang Y Luo P Jiang A. iMLGAM: Integrated Machine Learning and Genetic Algorithm-driven Multiomics analysis for pan-cancer immunotherapy response prediction. Imeta. (2025) 4:e70011. doi: 10.1002/imt2.70011 40236779 PMC11995183

[13] Niu X Li G Kahlert UD Ding L Zheng J Li C. Integrative disulfidptosis-based risk assessment for prognostic stratification and immune profiling in glioma. J Cell Mol Med. (2025) 29:e70429. doi: 10.1111/jcmm.70429 39993959 PMC11850091

[14] Zhang N Zhang H Liu Z Dai Z Wu W Zhou R. An artificial intelligence network-guided signature for predicting outcome and immunotherapy response in lung adenocarcinoma patients based on 26 machine learning algorithms. Cell Prolif. (2023) 56:e13409. doi: 10.1111/cpr.13409 36822595 PMC10068958

[15] Liu Q Long Q Zhao J Wu W Lin Z Sun W. Cold-induced reprogramming of subcutaneous white adipose tissue assessed by single-cell and single-nucleus RNA sequencing. Research (Wash D C). (2023) 6:182. doi: 10.34133/research.0182 PMC10308956 37398933

[16] Qi R Zou Q. Trends and potential of machine learning and deep learning in drug study at single-cell level. Research (Wash D C). (2023) 6:50. doi: 10.34133/research.0050 PMC10013796 36930772

[17] Yan R Zhang H Ma Y Lin R Zhou B Zhang T. Discovery of muscle-tendon progenitor subpopulation in human myotendinous junction at single-cell resolution. Research (Wash D C). (2022) 2022:9760390. doi: 10.34133/2022/9760390 36267539 PMC9555880

[18] Lu J Ren J Liu J Lu M Cui Y Liao Y. High-resolution single-cell transcriptomic survey of cardiomyocytes from patients with hypertrophic cardiomyopathy. Cell Prolif. (2024) 57:e13557. doi: 10.1111/cpr.13557 37766635 PMC10905351

[19] Chen Y Li C Wang N Wu Z Zhang J Yan J. Identification of LINC00654-NINL regulatory axis in diffuse large B-cell lymphoma in silico analysis. Front Oncol. (2022) 12:883301. doi: 10.3389/fonc.2022.883301 PMC9204339 35719990

[20] Noorbakhsh VS Ebrahimzadeh F Akbari OM Khalili S Almasi F Mosaddeghi HR. Potential promising anticancer applications of beta-glucans: a review. Biosci Rep. (2024) 44. doi: 10.1042/BSR20231686 PMC10776902 38088444

[21] Ma R Zhou X Zhai X Wang C Hu R Chen Y. Single-cell RNA sequencing reveals immune cell dysfunction in the peripheral blood of patients with highly aggressive gastric cancer. Cell Prolif. (2024) 57:e13591. doi: 10.1111/cpr.13591 38319150 PMC11056698

[22] Wang Z Dai Z Zhang H Zhang N Liang X Peng L. Comprehensive analysis of pyroptosis-related gene signatures for glioblastoma immune microenvironment and target therapy. Cell Prolif. (2023) 56:e13376. doi: 10.1111/cpr.13376 36681858 PMC9977674

[23] Ye B Jiang A Liang F Wang C Liang X Zhang P. Navigating the immune landscape with plasma cells: A pan-cancer signature for precision immunotherapy. Biofactors. (2025) 51:e2142. doi: 10.1002/biof.v51.1 39495620

[24] Chen L He Y Duan M Yang T Chen Y Wang B. Exploring NUP62’s role in cancer progression, tumor immunity, and treatment response: insights from multi-omics analysis. Front Immunol. (2025) 16:1559396. doi: 10.3389/fimmu.2025.1559396 40098960 PMC11911477

[25] Duan Y Wu Y Tian J Yin Y Yuan Z Zhu W. Elucidation of the mechanism Underlying the promotion of ferroptosis and enhanced antitumor immunity by citrus polymethoxyflavones in CRC cells. Front Pharmacol. (2025) 16:1571178. doi: 10.3389/fphar.2025.1571178 40290432 PMC12021823

[26] Wu X Lu W Xu C Jiang C Zhuo Z Wang R. Macrophages phenotype regulated by IL-6 are associated with the prognosis of platinum-resistant serous ovarian cancer: integrated analysis of clinical trial and omics. J Immunol Res. (2023) 2023:6455704. doi: 10.1155/2023/6455704 37124547 PMC10132904

[27] Yu Y Huang Y Li C Ou S Xu C Kang Z. Clinical value of M1 macrophage-related genes identification in bladder urothelial carcinoma and *in vitro* validation. Front Genet. (2022) 13:1047004. doi: 10.3389/fgene.2022.1047004 36468020 PMC9709473

[28] Zhao Z Ding Y Tran LJ Chai G Lin L. Innovative breakthroughs facilitated by single-cell multi-omics: manipulating natural killer cell functionality correlates with a novel subcategory of melanoma cells. Front Immunol. (2023) 14:1196892. doi: 10.3389/fimmu.2023.1196892 37435067 PMC10332463

[29] Lin Z Wang F Yin R Li S Bai Y Zhang B. Single-cell RNA sequencing and immune microenvironment analysis reveal PLOD2-driven Malignant transformation in cervical cancer. Front Immunol. (2024) 15:1522655. doi: 10.3389/fimmu.2024.1522655 39840054 PMC11747275

[30] Li X Lin Z Zhao F Huang T Fan W Cen L. Unveiling the cellular landscape: insights from single-cell RNA sequencing in multiple myeloma. Front Immunol. (2024) 15:1458638. doi: 10.3389/fimmu.2024.1458638 39281682 PMC11392786

[31] Jiang H Yu D Yang P Guo R Kong M Gao Y. Revealing the transcriptional heterogeneity of organ-specific metastasis in human gastric cancer using single-cell RNA Sequencing. Clin Transl Med. (2022) 12:e730. doi: 10.1002/ctm2.v12.2 35184420 PMC8858624

[32] Zhao F Jiang X Li Y Huang T Xiahou Z Nie W. Characterizing tumor biology and immune microenvironment in high-grade serous ovarian cancer via single-cell RNA sequencing: insights for targeted and personalized immunotherapy strategies. Front Immunol. (2024) 15:1500153. doi: 10.3389/fimmu.2024.1500153 39896800 PMC11782144

[33] Hou M Zhao Z Li S Zhang Z Li X Zhang Y. Single-cell analysis unveils cell subtypes of acral melanoma cells at the early and late differentiation stages. J Cancer. (2025) 16:898–916. doi: 10.7150/jca.102045 39781353 PMC11705046

[34] Lin L Zou J Pei S Huang W Zhang Y Zhao Z. Germinal center B-cell subgroups in the tumor microenvironment cannot be overlooked: Their involvement in prognosis, immunotherapy response, and treatment resistance in head and neck squamous carcinoma. Heliyon. (2024) 10:e37726. doi: 10.1016/j.heliyon.2024.e37726 39391510 PMC11466559

[35] Zhang Y Zhao Z Huang W Kim BS Lin L Li X. Pan-cancer single-cell analysis revealing the heterogeneity of cancer-associated fibroblasts in skin tumors. Curr Gene Ther. (2024). doi: 10.2174/0115665232331353240911080642 39323331

[36] Wu F Fan J He Y Xiong A Yu J Li Y. Single-cell profiling of tumor heterogeneity and the microenvironment in advanced non-small cell lung cancer. Nat Commun. (2021) 12:2540. doi: 10.1038/s41467-021-22801-0 33953163 PMC8100173

[37] An Y Zhao F Jia H Meng S Zhang Z Li S. Inhibition of programmed cell death by melanoma cell subpopulations reveals mechanisms of melanoma metastasis and potential therapeutic targets. Discov Oncol. (2025) 16:62. doi: 10.1007/s12672-025-01789-9 39832036 PMC11747064

[38] Jin W Zhang Y Zhao Z Gao M. Developing targeted therapies for neuroblastoma by dissecting the effects of metabolic reprogramming on tumor microenvironments and progression. Theranostics. (2024) 14:3439–69. doi: 10.7150/thno.93962 PMC11209723 38948053

[39] Nie W Zhao Z Liu Y Wang Y Zhang J Hu Y. Integrative single-cell analysis of cardiomyopathy identifies differences in cell stemness and transcriptional regulatory networks among fibroblast subpopulations. Cardiol Res Pract. (2024) 2024:3131633. doi: 10.1155/2024/3131633 38799173 PMC11127766

[40] Li H Bian Y Xiahou Z Zhao Z Zhao F Zhang Q. The cellular signaling crosstalk between memory B cells and tumor cells in nasopharyngeal carcinoma cannot be overlooked: Their involvement in tumor progression and treatment strategy is significant. J Cancer. (2025) 16:288–314. doi: 10.7150/jca.101420 39744570 PMC11660138

[41] Huang W Kim BS Zhang Y Lin L Chai G Zhao Z. Regulatory T cells subgroups in the tumor microenvironment cannot be overlooked: Their involvement in prognosis and treatment strategy in melanoma. Environ Toxicol. (2024) 39:4512–30. doi: 10.1002/tox.v39.10 38530049

[42] Ge Q Zhao Z Li X Yang F Zhang M Hao Z. Deciphering the suppressive immune microenvironment of prostate cancer based on CD4+ regulatory T cells: Implications for prognosis and therapy prediction. Clin Transl Med. (2024) 14:e1552. doi: 10.1002/ctm2.v14.1 38239097 PMC10797244

[43] Ding Y Zhao Z Cai H Zhou Y Chen H Bai Y. Single-cell sequencing analysis related to sphingolipid metabolism guides immunotherapy and prognosis of skin cutaneous melanoma. Front Immunol. (2023) 14:1304466. doi: 10.3389/fimmu.2023.1304466 38077400 PMC10701528

[44] Zhou Y Yang D Yang Q Lv X Huang W Zhou Z. Single-cell RNA landscape of intratumoral heterogeneity and immunosuppressive microenvironment in advanced osteosarcoma. Nat Commun. (2020) 11:6322. doi: 10.1038/s41467-020-20059-6 33303760 PMC7730477

[45] Korsunsky I Millard N Fan J Slowikowski K Zhang F Wei K. Fast, sensitive and accurate integration of single-cell data with Harmony. Nat Methods. (2019) 16:1289–96. doi: 10.1038/s41592-019-0619-0 PMC6884693 31740819

[46] He Y Luo Z Nie X Du Y Sun R Sun J. An injectable multi-functional composite bioactive hydrogel for bone regeneration via immunoregulatory and osteogenesis effects. Adv Composites Hybrid Mater. (2025) 8:128. doi: 10.1007/s42114-025-01213-4

[47] Wang J Zhao F Zhang Q Sun Z Xiahou Z Wang C. Unveiling the NEFH+ Malignant cell subtype: Insights from single-cell RNA sequencing in prostate cancer progression and tumor microenvironment interactions. Front Immunol. (2024) 15:1517679. doi: 10.3389/fimmu.2024.1517679 39759507 PMC11695424

[48] Ni G Sun Y Jia H Xiahou Z Li Y Zhao F. MAZ-mediated tumor progression and immune evasion in hormone receptor-positive breast cancer: Targeting tumor microenvironment and PCLAF+ subtype-specific therapy. Transl Oncol. (2025) 52:102280. doi: 10.1016/j.tranon.2025.102280 39805182 PMC11780959

[49] Zhao ZJ Zheng RZ Wang XJ Li TQ Dong XH Zhao CY. Integrating lipidomics and transcriptomics reveals the crosstalk between oxidative stress and neuroinflammation in central nervous system demyelination. Front Aging Neurosci. (2022) 14:870957. doi: 10.3389/fnagi.2022.870957 35547618 PMC9083465

[50] Zhao ZJ Wei DP Zheng RZ Peng T Xiao X Li FS. The gene coexpression analysis identifies functional modules dynamically changed after traumatic brain injury. Comput Math Methods Med. (2021) 2021:5511598. doi: 10.1155/2021/5511598 33953790 PMC8068551

[51] Li XY Zhao ZJ Wang JB Shao YH Hui-Liu You JX. m7G methylation-related genes as biomarkers for predicting overall survival outcomes for hepatocellular carcinoma. Front Bioeng Biotechnol. (2022) 10:849756. doi: 10.3389/fbioe.2022.849756 35620469 PMC9127183

[52] Feng X Luo Z Zhang W Wan R Chen Y Li F. Zn-DHM nanozymes enhance muscle regeneration through ROS scavenging and macrophage polarization in volumetric muscle loss revealed by single-cell profiling. Adv Funct Mater. (2025). doi: 10.1002/adfm.202506476

[53] Yu G Wang LG Han Y He QY. clusterProfiler: an R package for comparing biological themes among gene clusters. Omics. (2012) 16:284–7. doi: 10.1089/omi.2011.0118 PMC3339379 22455463

[54] Zhao Z Li T Dong X Wang X Zhang Z Zhao C. Untargeted metabolomic profiling of cuprizone-induced demyelination in mouse corpus callosum by UPLC-orbitrap/MS reveals potential metabolic biomarkers of CNS demyelination disorders. Oxid Med Cell Longev. (2021) 2021:7093844. doi: 10.1155/2021/7093844 34567412 PMC8457991

[55] Sun L Shao W Lin Z Lin J Zhao F Yu J. Single-cell RNA sequencing explored potential therapeutic targets by revealing the tumor microenvironment of neuroblastoma and its expression in cell death. Discov Oncol. (2024) 15:409. doi: 10.1007/s12672-024-01286-5 39235657 PMC11377405

[56] Zhao F Hong J Zhou G Huang T Lin Z Zhang Y. Elucidating the role of tumor-associated ALOX5+ mast cells with transformative function in cervical cancer progression via single-cell RNA sequencing. Front Immunol. (2024) 15:1434450. doi: 10.3389/fimmu.2024.1434450 39224598 PMC11366577

[57] Shao W Lin Z Xiahou Z Zhao F Xu J Liu X. Single-cell RNA sequencing reveals that MYBL2 in Malignant epithelial cells is involved in the development and progression of ovarian cancer. Front Immunol. (2024) 15:1438198. doi: 10.3389/fimmu.2024.1438198 39136009 PMC11317301

[58] Jin S Guerrero-Juarez CF Zhang L Chang I Ramos R Kuan CH. Inference and analysis of cell-cell communication using CellChat. Nat Commun. (2021) 12:1088. doi: 10.1038/s41467-021-21246-9 33597522 PMC7889871

[59] Zhou W Lin Z Tan W. Deciphering the molecular landscape: integrating single-cell transcriptomics to unravel myofibroblast dynamics and therapeutic targets in clear cell renal cell carcinomas. Front Immunol. (2024) 15:1374931. doi: 10.3389/fimmu.2024.1374931 38562930 PMC10982338

[60] Liu P Xing N Xiahou Z Yan J Lin Z Zhang J. Unraveling the intricacies of glioblastoma progression and recurrence: insights into the role of NFYB and oxidative phosphorylation at the single-cell level. Front Immunol. (2024) 15:1368685. doi: 10.3389/fimmu.2024.1368685 38510250 PMC10950940

[61] Lee DY Chun JN Cho M So I Jeon JH. Emerging role of E2F8 in human cancer. Biochim Biophys Acta Mol Basis Dis. (2023) 1869:166745. doi: 10.1016/j.bbadis.2023.166745 37164180

[62] Liu K Wang L Lou Z Guo L Xu Y Qi H. E2F8 exerts cancer-promoting effects by transcriptionally activating RRM2 and E2F8 knockdown synergizes with WEE1 inhibition in suppressing lung adenocarcinoma. Biochem Pharmacol. (2023) 218:115854. doi: 10.1016/j.bcp.2023.115854 37863324

[63] Zhou XH Xu H Xu C Yan YC Zhang LS Sun Q. Hepatocellular carcinoma-derived exosomal miRNA-761 regulates the tumor microenvironment by targeting the SOCS2/JAK2/STAT3 pathway. World J Emerg Med. (2022) 13:379–85. doi: 10.5847/wjem.j.1920-8642.2022.089 PMC9420661 36119773

[64] Wan R Pan L Wang Q Shen G Guo R Qin Y. Decoding gastric cancer: machine learning insights into the significance of COMMDs family in immunotherapy and diagnosis. J Cancer. (2024) 15:3580–95. doi: 10.7150/jca.94360 PMC11134438 38817875

[65] Wang Q Zheng C Hou H Bao X Tai H Huang X. Interplay of sphingolipid metabolism in predicting prognosis of GBM patients: towards precision immunotherapy. J Cancer. (2024) 15:275–92. doi: 10.7150/jca.89338 PMC10751665 38164288

[66] Liu Y Wang S Liu J Su M Diao X Liang X. Characteristics of vaginal microbiota in various cervical intraepithelial neoplasia: a cross-sectional study. J Transl Med. (2023) 21:816. doi: 10.1186/s12967-023-04676-5 37974192 PMC10652498

[67] Kyrgiou M Athanasiou A Kalliala I Paraskevaidi M Mitra A Martin-Hirsch PP. Obstetric outcomes after conservative treatment for cervical intraepithelial lesions and early invasive disease. Cochrane Database Syst Rev. (2017) 11:CD012847. doi: 10.1002/14651858.CD012847 29095502 PMC6486192

[68] Uuskula-Reimand L Wilson MD. Untangling the roles of TOP2A and TOP2B in transcription and cancer. Sci Adv. (2022) 8:eadd4920. doi: 10.1126/sciadv.add4920 36322662 PMC9629710

[69] Wiegard A Kuzin V Cameron DP Grosser J Ceribelli M Mehmood R. Topoisomerase 1 activity during mitotic transcription favors the transition from mitosis to G1. Mol Cell. (2021) 81:5007–24. doi: 10.1016/j.molcel.2021.10.015 34767771

[70] Zhu J Fan Y Xiong Y Wang W Chen J Xia Y. Delineating the dynamic evolution from preneoplasia to invasive lung adenocarcinoma by integrating single-cell RNA sequencing and spatial transcriptomics. Exp Mol Med. (2022) 54:2060–76. doi: 10.1038/s12276-022-00896-9 PMC9722784 36434043

[71] Xu P Yang J Chen Z Zhang X Xia Y Wang S. N6-methyladenosine modification of CENPF mRNA facilitates gastric cancer metastasis via regulating FAK nuclear export. Cancer Commun (Lond). (2023) 43:685–705. doi: 10.1002/cac2.12443 37256823 PMC10259669

[72] Liu ZL Chen N Li R Ma YJ Qiayimaerdan A Ma CL. WGCNA reveals a biomarker for cancer-associated fibroblasts to predict prognosis in cervical cancer. J Chin Med Assoc. (2024) 87:885–97. doi: 10.1097/JCMA.0000000000001129 PMC13610161 38946034

[73] Kwon EJ Lee HR Lee JH Seo C Ha M Roh J. Identification of differentially expressed genes and pathways for risk stratification in HPV-associated cancers governing different anatomical sites. Front Biosci (Landmark Ed). (2022) 27:2. doi: 10.31083/j.fbl2701002 35090306

[74] Khan MA Khan P Ahmad A Fatima M Nasser MW. FOXM1: A small fox that makes more tracks for cancer progression and metastasis. Semin Cancer Biol. (2023) 92:1–15. doi: 10.1016/j.semcancer.2023.03.007 36958703 PMC10199453

[75] Liang H Lu Q Yang J Yu G. Supramolecular biomaterials for cancer immunotherapy. Research (Wash D C). (2023) 6:211. doi: 10.34133/research.0211 PMC10496790 37705962

[76] Danishuddin, Haque MA Khan S Kim JJ Ahmad K. Molecular landscape of bladder cancer: key genes, transcription factors, and drug interactions. Int J Mol Sci. (2024) 25(20):10997. doi: 10.3390/ijms252010997 39456780 PMC11507096

[77] Ershov P Poyarkov S Konstantinova Y Veselovsky E Makarova A. Transcriptomic signatures in colorectal cancer progression. Curr Mol Med. (2023) 23:239–49. doi: 10.2174/1566524022666220427102048 35490318

